# Anti-Inflammatory Effect of IL-37-Producing T-Cell Population in DSS-Induced Chronic Inflammatory Bowel Disease in Mice

**DOI:** 10.3390/ijms19123884

**Published:** 2018-12-05

**Authors:** Zhangbo Chen, Shijun Wang, Lingyun Li, Zhong Huang, Ke Ma

**Affiliations:** 1 Department of Immunology, Shenzhen University School of Medicine, Nanhai Ave. 3688, Shenzhen 518060, China; chzb@szu.edu.cn (Z.C.); cat_love_guai@163.com (L.L.); 2 Institute of Biological Therapy, Shenzhen University, Nanhai Ave 3688, Shenzhen 518060, China; 3 Shandong Co-Innovation Center of Classic TCM formula, Shandong University of Traditional Chinese Medicine, Jinan 250355, China; wsj@sdutcm.edu.cn

**Keywords:** T lymphocyte, chronic IBD, IL-37

## Abstract

Inflammatory bowel disease (IBD) is a chronic inflammatory disease that is thought to arise in part from abnormal adaptive immune responses against intestinal microbiota. T lymphocytes play significant roles in triggering mucosal inflammation and/or maintaining gut immune homeostasis. It has been demonstrated that IL-37 expresses in a variety of cells and exerts a protective function involved in both innate immunity and adaptive immunity. In the present study, a population of IL-37-producing T-cells was detected in the spleen and mesenteric lymph nodes (MLNs) in IL-37^+/+^ mice after dextran sodium sulfate (DSS) induction. Adoptive transfer of the T-cells from the spleen of IL-37^+/+^ mice following DSS treatment partly recovered the body weight, improved the disease activity index (DAI) and macroscopic damage score, and attenuated the intestinal inflammation. In addition, colon shortening, an indirect marker of inflammation, was decreased, consistent with the decreased IFN-γ level and the increased IL-10 level in the colonic tissue. Collectively, our data uncovered a subset of T-lymphocytes expressing IL-37, which represents a potent regulation of immunity and serves as the protective role in chronic IBD.

## 1. Introduction

The inflammatory bowel diseases (IBD), Crohn’s disease (CD) and ulcerative colitis (UC), are chronic inflammatory disorders of the intestine with a relapsing-remitting clinical course [1,2]. The aetiology of IBD is unclear. T lymphocytes, which represent the key cell population in intestinal lamina propria and epithelium, play important roles during active immune responses and maintenance of gut immune homeostasis [3,4]. Because of the heterogeneity of this cell, the precise role of T lymphocyte in IBD is not completely understood. IBD was previously thought to be linked with Th1 cells in CD or Th2 cells in UC [5,6]. However, whether this paradigm could distinguish CD from UC is controversial [7]. More recently, Th17 cells which are capable of producing IL (interleukin)-17A and other cytokines have been heavily implicated in the pathogenesis of IBD [7]. Although a pro-inflammatory role in mucosal immune responses has been ascribed to Th17 cells, several different studies in experimental colitis have reported protective effects of Th17-type cytokines in the intestinal tract [8,9]. In addition, regulatory T-cells (Treg cells), which are characterized by the expression of the Foxp3 transcription factor, provide an essential control of excessive effector T-cell responses to enterobacterial antigens in IBD [10,11].

IL-37, a member of the IL-1 family, has been considered as a fundamental suppressor of innate immunity [12]. Five splice variants (IL-37a–e) have been identified in humans but not in mice [12]. The inhibitory activity of IL-37 was demonstrated in both human and animal studies such as lipopolysaccharide (LPS)-induced shock, acute myocardial infarction, and spinal cord contusion [12,13,14]. Recent research has found that IL-37 not only participates in innate immunity, but also emerges as an inhibitor of adaptive immunity. Luo et al. indicated that dendritic cells expressing IL-37 impaired the activation of effector T-cell responses and induced Treg cells in the mouse model of contact hypersensitivity [15]. Moreover, IL-37 has been shown to restrain inflammation and Th2/Th17 cell activation in a murine model of invasive pulmonary aspergillosis, suggesting that IL-37 mediates the inhibition effect on adaptive immune response [16]. The role of IL-37 in IBD is not well understood. Some clinical research groups reported the intestinal expression of IL-37 in the inflamed bowels of patients with IBD, the levels of which are associated with the degree of local mucosal inflammation [17,18]. In addition, McNamee et al. showed that the severity of the intestinal inflammation was lower in IL-37tg mice as compared to wild-type (WT) controls, suggesting that IL-37 acts as an anti-inflammatory cytokine that could down-regulate colitis [19].

In the present study, we investigated the role of IL-37 with a transgenic mouse strain that expresses human IL-37 in a murine model of colitis resulting from repeated cycles of dextran sodium sulfate (DSS) administration that mimics the relapsing nature of the IBD. We determined the IL-37 expression in gut mucosal sites, and the effect of IL-37 on the development of chronic colitis. Furthermore, we detected the population of T-cells which expressed IL-37 in the spleen and mesenteric lymph nodes (MLNs). The anti-inflammatory impact of IL-37-producing T-cell population was also tested using an adoptive transfer approach.

## 2. Results

### 2.1. Transgenic IL-37 Expression Alleviates DSS (Dextran Sodium Sulfate)-Induced Colitis in Mice

**Figure 1:**
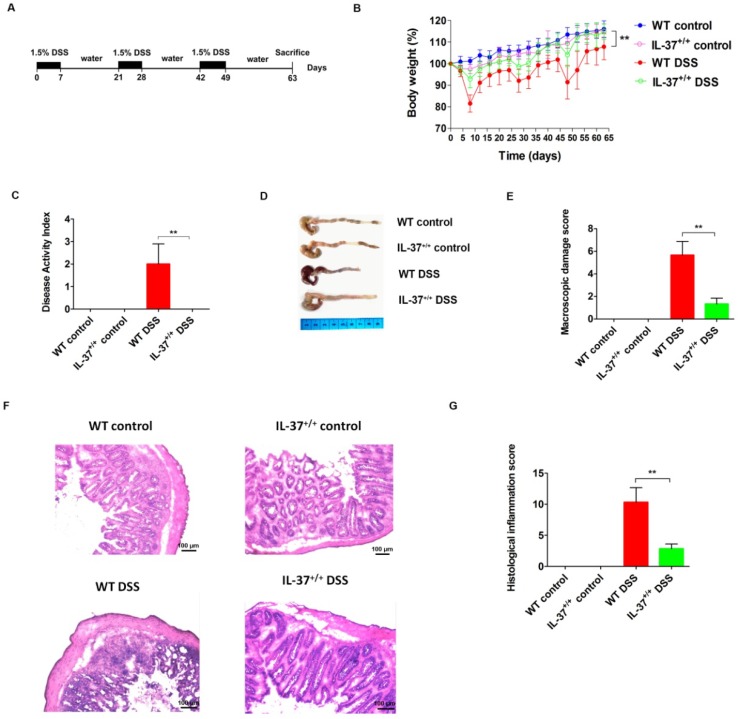
IL-37 reduces dextran sodium sulfate (DSS)-induced chronic colitis in mice. (**A**) Chronic colitis was induced by DSS for seven days followed by a recovery period of two weeks with normal drinking water. This was repeated three times, with sacrifice at day 63. Control mice received normal drinking water throughout. (**B**) Relative weight curves of wild-type (WT) and IL-37^+/+^ mice in chronic colitis. The statistical significance between the relative weights of DSS-exposed IL-37^+/+^ versus WT mice at the end of the experiment is shown. (**C**) The disease activity index (DAI) was determined at day 63 of the study period, based on body weight loss (0, none; 1, 1–5%; 2, 5–10%; 3, 10–20%; 4, >20%), stool consistency (0, normal; 2, loose stool; 4, diarrhea), and stool blood (0, negative; 2, fecal occult blood test positive; 4, gross bleeding). (**D**) Representative pictures of the colon from indicated treatment cohorts. (**E**) Macroscopic damage score of the colon in the four groups. The macroscopic damage score was calculated based on the extent of inflammation along the colon (one point for each centimeter of moderate inflammation or two points for each centimeter of severe inflammation evaluated by the thickness of the colon), colonic mesenterial adhesion (0, none; 1, mild to moderate; 2, moderate to severe) and colonic hyperaemia (0, none; 1, present). (**F**) Representative haematoxylin and eosin (H&E) staining of colon sections of WT and IL-37^+/+^ mice after administration of DSS. (**G**) Histological inflammation score. This score comprises the sum of architecture of the bowel, infiltration of neutrophils and mononuclear cells, gobleT-cell depletion, and epithelial cell erosion. Three sections per animal were evaluated. Data are means ± SEM (*n* = 6). ** *p* < 0.01.

The experimental set-up for induction of chronic colitis is shown in Figure 1A. All mice experienced weight loss during the first cycle of DSS. However, IL37^+/+^ mice recovered more quickly after the first week of DSS administration compared with WT mice, and lost less weight during the second and third cycles of DSS administration, resulting in an obviously increased relative weight compared with WT mice after the induction of chronic colitis (*p* = 0.003) (Figure 1B). In line with this, the DAI was lower in IL37^+/+^ mice compared with WT mice after repeated cycles of DSS administration (*p* < 0.01) (Figure 1C). The colon of WT mice (7.5 ± 0.3 cm) was significantly shorter than the colon of IL37^+/+^ mice (8.6 ± 0.3 cm) after the induction of chronic colitis (*p* < 0.01) (Figure 1D). In addition, IL-37^+/+^ mice exhibited a decreased macroscopic damage score compared with WT mice after chronic administration of DSS (Figure 1E). The colon histology of WT mice treated with DSS showed extensive leukocyte infiltrates (Figure 1F). In contrast, IL-37 expression reversed the increase in cellular infiltrates after DSS treatment (Figure 1F). The histological scores significantly decreased after the induction of chronic colitis in IL37^+/+^ mice compared with WT mice (Figure 1G).

### 2.2. IL-37 Expression Is Inducible and Inhibits the Colonic Proinflammatory Cytokines

**Figure 2:**
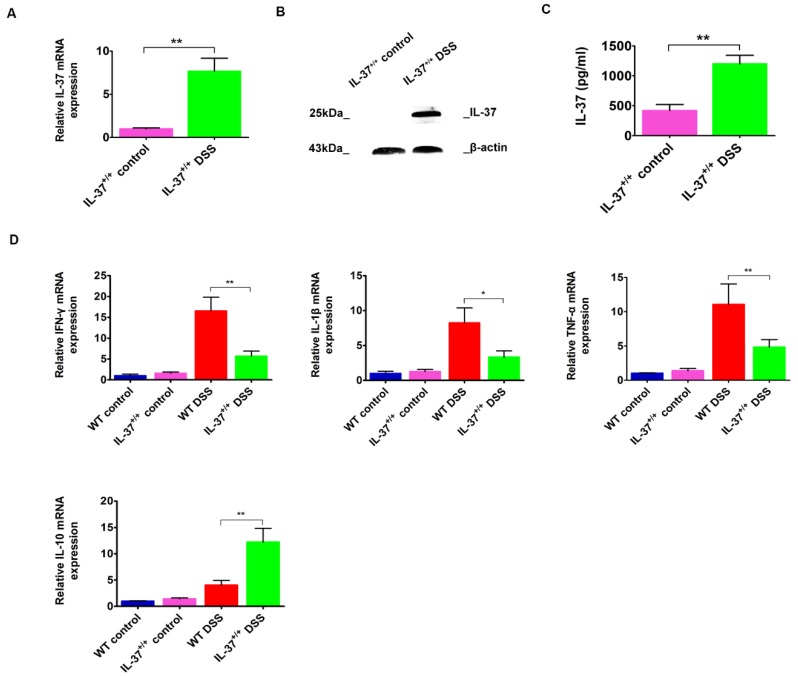
The gene and protein expression of IL-37 and the expression of colonic IFN-γ, IL-1β, TNF-α, and IL-10 following DSS induction in IL-37^+/+^ mice. (**A**,**B**) Expressions of IL-37 mRNAs and protein levels within colonic tissues following DSS induction in IL-37^+/+^ mice, respectively. (**C**) Expressions of IL-37 proteins in serum following DSS induction in IL-37^+/+^ mice. (**D**) Expressions of IFN-γ, IL-1β, TNF-α, and IL-10 mRNAs within colonic tissues following DSS induction in IL-37^+/+^ mice. Data are means ± SEM (*n* = 6). * *p* < 0.05, ** *p* < 0.01.

We next examined the IL-37 expression in colonic tissues after the treatment of DSS. IL-37 mRNA and protein were not detected in WT mice. Although the baseline values were minimal, IL-37 mRNA expression was significantly increased following DSS administration, reaching approximately a 7.5-fold increase on day 63 in IL37^+/+^ mice (Figure 2A). Similarly, the protein levels for IL-37 were almost undetectable in the IL-37^+/+^ control group but were increased after the treatment of DSS-induced colitis (Figure 2B). In addition, our results showed that IL-37 levels in the serum were elevated after DSS treatment in IL37^+/+^ mice (Figure 2C).

Moreover, we found that the mRNA levels of inflammatory factors such as IFN-γ, IL-1β, and TNF-α, were reduced in IL-37^+/+^ mice compared with WT mice after DSS induction (Figure 2D). In contrast, the mRNA levels of IL-10, an anti-inflammation factor, were elevated after the induction of DSS (Figure 2D). This, together with the reduction in colonic immune cells, indicates that IL-37 expression limits the inflammatory state of the mice.

### 2.3. IL-37-Producing T-Cell Population in Chronic Colitis

**Figure 3:**
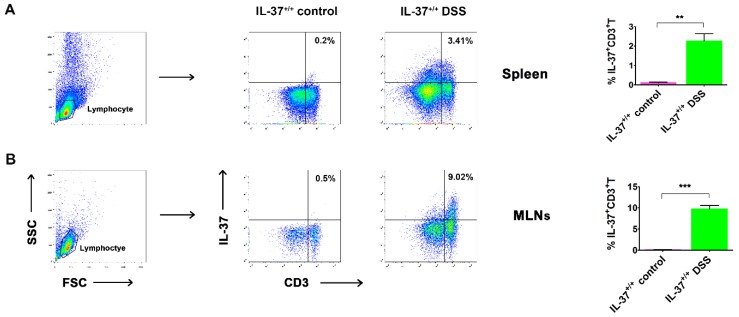
The IL-37-producing T-cells in the spleen and mesenteric lymph nodes (MLNs) in IL-37^+/+^ mice. Representative dot plots showing the percentage of IL-37^+^ cells within the CD3^+^ T-cell population in the (**A**) spleen and (**B**) MLNs of DSS-treated mice at day 63, respectively. Data are means ± SEM (*n* = 6). ** *p* < 0.01, *** *p* < 0.001.

Previous reports suggest that IBD is related to an excessive activation of effector T-cells and/or alteration of Treg immunosuppressive properties [3,20]. We examined here whether the expression of IL-37, an inhibitor of inflammatory and immune responses, could be detected on T-cells after the induction of chronic colitis. Lymphocytes were gated according to FSC and SSC, and the percentage of IL-37 was shown as CD3 (Figure 3A). Although very low expression of IL-37 was detected on CD3^+^ T-cells in the spleen (0.22 ± 0.06%) of mice in the IL-37^+/+^ control group, a significant increase of it was detected following DSS induction (3.47 ± 1.22%) (Figure 3A,B). Similar findings were observed in MLNs where the expression of IL-37 on CD3^+^ T-cells was remarkably increased after the induction of DSS (0.48 ± 0.12% versus 9.75 ± 2.71%) (Figure 3A,B).

### 2.4. Anti-Inflammatory Role of IL-37-Producing T-Cells in DSS-Induced Colitis

**Figure 4:**
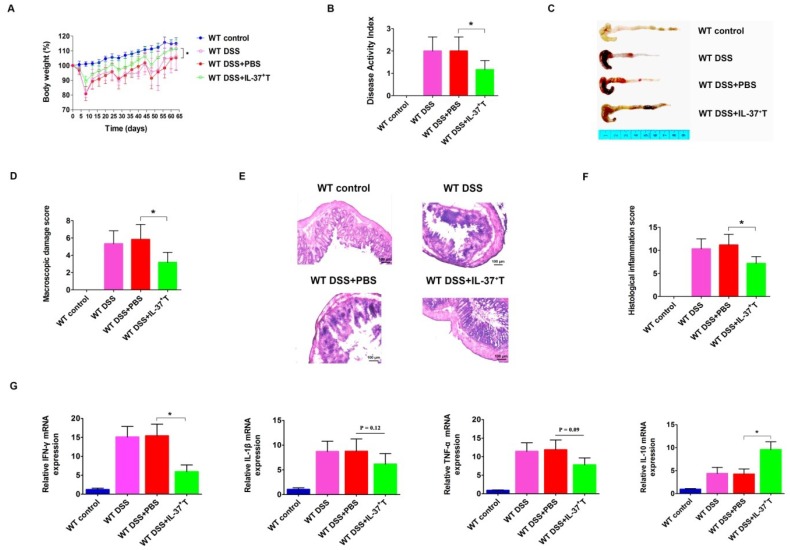
IL-37^+^ T-cells play a protective role in DSS-induced chronic colitis in mice. (**A**) Relative weight curves of WT mice, DSS-induced WT mice, DSS-induced WT mice treated with PBS, and DSS-induced WT mice injected with IL-37^+^ T-cells in chronic colitis. The statistical significance between the relative weights of DSS-induced mice injected with IL-37^+^ T-cells versus DSS-induced mice treated with PBS at the end of the experiment is shown. (**B**) The disease activity index (DAI) was determined at day 63 of the study period, based on body weight loss (0, none; 1, 1–5%; 2, 5–10%; 3, 10–20%; 4, >20%), stool consistency (0, normal; 2, loose stool; 4, diarrhea), and stool blood (0, negative; 2, fecal occult blood test positive; 4, gross bleeding). (**C**) Representative pictures of the colon from indicated treatment cohorts. (**D**) Macroscopic damage score of the colon in the four groups. (**E**) Representative haematoxylin and eosin (H&E) staining of colon sections of mice. (**F**) Histological inflammation score. This score comprises the sum of architecture of the bowel, infiltration of neutrophils and mononuclear cells, gobleT-cell depletion, and epithelial cell erosion. Three sections per animal were evaluated. (**G**) Expressions of IFN-γ, IL-1β, TNF-α, and IL-10 mRNAs within colonic tissues in the four groups. Total RNA with the RNAiso Plusi Kit. cDNAs were synthesized by using the RevertAid First Strand cDNA Synthesis Kit according to the manufacturer’s instructions. qPCR amplification reactions were prepared with the SYBR Green PCR Kit and performed using the CFX96 Real-Time System. Data are means ± SEM (*n* = 6). * *p* < 0.05.

As shown in previous studies, IL-37-expressing cells exert protective effects on several established animal disease models [21,22]. Therefore, we examined whether IL-37-producing T-cells could improve the colitis activity in our DSS-induced mouse model. As the IL-37b pIRES plasmid was driven by a CMV promoter, the IL-37 protein was supposed to be expressed on all karyotes. We measured the optimal concentration of CD3^+^ T-cells by several preliminary experiments, and then treated the mice on day 0 with 4 × 10^6^ freshly isolated CD3^+^ T-cells. Indeed, the adoptive transfer of CD3^+^ T-cells from the spleen was effective in inhibiting weight loss during DSS-induced chronic colitis. In the WT DSS + PBS group, mice weighed 105.2 ± 7.1% of the original body weight at day 63 (Figure 4A). Mice in the WT DSS + CD3^+^ T group weighed 111.4 ± 9.6% of the original body weight. In line with this, the DAI of IL-37-producing T-cell-treated mice was lower than that of the WT DSS + PBS group mice (Figure 4B). Compared to the WT DSS + PBS mice, mice that received IL-37-expressing T-cells showed less severe inflammation (as the shortened colon lengths of the WT DSS + IL-37^+^ T mice were lower than the WT DSS + PBS group; WT DSS + IL-37^+^ T group, 8.3 ± 0.3 cm vs. WT DSS + PBS group 7.4 ± 0.3 cm, *p* < 0.05) (Figure 4C). Moreover, transferring IL-37-producing T-cells also improved the macroscopic damage score (Figure 4D). Histological analysis of the colon also revealed that the intestinal inflammation of DSS-treated mice that were injected with IL-37-expressing T-cells was lower than those injected with PBS (Figure 4E). The histopathological colitis score in the WT DSS + IL-37^+^ T mice was lower than the score of the WT DSS + PBS-treated mice (*p* < 0.05, Figure 4F).

We next investigated whether IL-37-producing T-cell treatment could alter the levels and expression of pro-inflammatory and anti-inflammatory cytokines in colon tissues. The mRNA levels of the pro-inflammatory cytokine IFN-γ was decreased in mice that received IL-37-producing T-cells, whereas the immunosuppressive cytokine IL-10 was increased (Figure 4G). Although there was no statistically significant difference, we observed that the expression of pro-inflammatory cytokines IL-1β and TNF-α appeared to be lower in the WT DSS + IL-37^+^ T-treated mice than in the WT DSS + PBS group mice (*p* = 0.07) (Figure 4G). These findings collectively indicate that the IL-37-producing T-cell population exerts an anti-inflammatory effect in this model of chronic colitis.

## 3. Discussion

The immunology of IBD conducts an imbalance between two types of T-cell populations: pro-inflammatory T-cells and Tregs. Traditionally, it was thought that CD was characterized predominantly by Th1 cells secreting IFN-γ and that UC was characterized by Th2 cells associated with IL-4, IL-5, and IL-13 production [23,24]. However, the Th1/Th2 paradigm has been questioned. More recently, the role of Th17 cells which are capable of producing IL-17 in the adaptive immune response in IBD has been described. Genome-wide association scan data indicate a role played by Th17 cells in IBD, and a combined blockade of IL-17A and IL-17F could obviously prevent the development of experimental colitis [25]. However, although Th17 cells have a pathogenic effect in intestine inflammation, other research in experimental colitis has reported a tissue-protective role of Th17-related cytokines in the gut. Kinugasa et al. suggest that IL-17A exerts its protective effects through promoting tight-junction formation by inducing the expression of claudins in intestinal epithelial cells, thereby increasing the mucosal barrier function [26]. Moreover, several studies have revealed that the Tregs, a subpopulation of T-cells which modulate the immune system, were involved in the control of colitis [20,27]. The pathogenic role of MHC class I-restricted CD8^+^ T-cells has also been demonstrated in murine colitis models and implied by observations in IBD patients. [28,29] Collectively, the reports above revealed the diversity of T subsets in the gut and that each of them has a distinct function in the pathogenesis of IBD.

IL-37, a member of the IL-1 family, was first discovered by in silico research in 2000 [30]. There are five transcripts for the human IL-37 gene, among which IL-37b is the most abundant isoform, and most likely to have biological function [31,32]. It has been observed that IL-37 was elevated in humans with inflammatory and autoimmune diseases, such as Graves’ disease, multiple sclerosis, and rheumatoid arthritis, although the concentrations of IL-37 in the circulation of healthy humans are low [33,34,35]. The elevated expression of this cytokine in human disease indicates that it may play a role in regulating the immune system. There is no IL-37 gene in mice. Nevertheless, numerous studies indicate that transgenic mice expressing the human IL-37 gene exhibit a wide range of anti-inflammatory features. In 2010, Marcel et al. demonstrated that mice with transgenic expression of IL-37 exhibit less hypothermia, metabolic acidosis, and dehydration, and are accompanied by an improved lung and kidney function as compared with control mice in a model of LPS-induced endotoxic shock [13]. Marina et al. also reported that IL-37-tg mice were protected from spinal cord contusion injury and exhibited increased myelin and neuronal sparing compared to WT mice which were subjected to the same injury [15]. Moreover, treatment of WT mice with recombinant human IL-37 has been shown to be protective in several animal models. In a mouse model of invasive pulmonary aspergillosis, recombinant IL-37 protected mice against acute lung injury by inhibiting inflammatory cell recruitment [17]. In addition, hydrodynamic injection of IL-37 alleviated renal ischemia-reperfusion injury and recovered kidney function by controlling the mononuclear cell infiltration in a mouse ischemic injury model. [36] The research on IL-37 levels in human diseases and on recombinant IL-37 being effective in suppressing inflammation in animal models indicates that IL-37 plays a significant role in limiting detrimental inflammation and preventing an exuberant immune response.

IL-37 has been found expressed in many cells including human peripheral blood mononuclear cells, macrophages, epithelial cells, activated B cells, and synovial cells [33]. Several studies indicated that the IL-37-expressing cells have a systemic or local anti-inflammation effect in different animal disease models. In 2011, McNamee et al. reported that hematopoietic cell-specific expression of IL-37 had protective effects on intestinal inflammation in their mouse model of acute DSS-induced colitis [37]. Recently, Geerte et al. showed that hematopoietic expression of IL-37 moderately reduced the inflammatory state, but did not affect the atherosclerotic lesion development and the macrophage content in hyperlipidemic LDLr-deficient mice under low-grade inflammatory conditions [22]. Moreover, in a later report, McCurdy et al. demonstrated that macrophage-expressed IL-37 reduced pro-inflammatory cytokine production and total plaque area, and more importantly, the development of atherosclerosis in atherosclerosis-prone Ldlr^−/−^ mice [23]. In the present study, we revealed that the IL-37 protein was expressed on T lymphocytes in IL-37^+/+^ mice in a model of DSS-induced chronic colitis, and more importantly, the IL-37-producing T subset exhibited an inhibitory effect on both the intestinal inflammation and the development of chronic colitis.

A better understanding of the immunological mechanisms that regulate intestinal inflammation could offer the possibility to develop novel drugs to control chronic intestinal inflammation. Further studies are needed to determine the precise mechanisms by which IL-37-producing T-cells attenuate the severity of intestinal injury. Nevertheless, this study may provide new insights and therapeutic approaches for preventing inflammatory bowel disease.

## 4. Materials and Methods

### 4.1. Mice

IL-37 transgenic mice (IL-37^+/+^) were constructed by the full length precursor cDNA of IL-37b isoform, and the cytomegalovirus promoter was used to drive the gene expression. IL-37b pIRES plasmids were injected into fertilized eggs of C57BL/6 mice and implanted into C57BL/6 females. Male IL-37 transgenic mouse founders were mated with C57BL/6 wild-type females. IL-37 transgenic mice were identified by genotyping PCRs, and Western blots were used to detect the protein expression on tails at 3–4 weeks of age. Male wild-type C57BL/6 mice, aged 8–10 weeks, were purchased from Guangdong Medical Laboratory Animal Center (Guangzhou, China) and used as controls. Animals were housed at the Health Science Center of Shenzhen University. To minimize the pain and distress, mice were maintained in barrier-, specific-pathogen free conditions with 16 h light:8 h dark at 22 °C in individually ventilated cages. There are no differences between the IL-37^+/+^ colony and wild-type mice including the growth, behavior and reproduction. The experimental protocols were approved by the Institutional Animal Care and Use Committee of Shenzhen University (AWEC201806175994, 17 June 2018).

### 4.2. DSS-Induced Chronic Colitis

Chronic colitis was induced by three cycles of 1.5% dextran sodium sulfate (DSS), and each cycle of DSS was defined as one week of DSS administration followed by a recovery period of two weeks with normal drinking water. Control mice (WT and IL37^+/+^) received normal drinking water throughout the duration of the experiment. All mice were sacrificed at day 63 (Figure 1A). The body weight of mice was monitored every 3–4 days after the initiation of DSS. The experiments were repeated at least three times.

### 4.3. Assessment of Disease Activity Index

The disease activity index (DAI) was measured based on the following parameters: consistency of stools (0, normal; 2, loose stool; 4, diarrhea), loss of body weight (0, none; 1, 1–5%; 2, 5–10%; 3, 10–20%; 4, >20%), and presence of gross blood in stools (0, negative; 2, fecal occult blood test positive; 4, gross bleeding) [37].

### 4.4. Evaluation of Colonic Inflammation and Histology

The entire colon was removed and cleaned. The length of colon was calculated from the caecum to the anus. One part of the colon was fixed in 4% formalin for histopathological evaluation. The other part of the colon was snap-frozen for quantitative real-time polymerase chain reaction (Real-Time PCR) and Western blot. The macroscopic damage score was calculated based on the extent of inflammation along the colon (one point for each centimeter of moderate inflammation or two points for each centimeter of severe inflammation evaluated by the thickness of the colon), colonic mesenterial adhesion (0, none; 1, mild to moderate; 2, moderate to severe) and colonic hyperaemia (0, none; 1, present) [38]. Histopathological evaluation of the colon was performed on paraffin embedded, 8 mm-thick longitudinal and transverse sections stained with haematoxylin and eosin (H&E). The histological inflammation score was calculated based on the sum of architecture of the bowel, infiltration of neutrophils and mononuclear cells, gobleT-cell depletion, and epithelial cell erosion [39]. Three sections per animal were evaluated and slides were scored by a pathologist blinded to the experimental condition.

### 4.5. RT-qPCR

Total RNA was isolated from the colon with the RNAiso Plusi Kit (Takara, TaKaRa, Dalian, China). After extracting RNA, the UV absorbance was used to measure RNA purity and quality. Three main wavelengths of interest are 260 nm, 280 nm and 230 nm. Absorbance at 260 nm was used to measure the amount of nucleic acid present in the sample. Absorbance at 280 nm are used to estimate the amount of protein in the sample. Absorbance at 230 nm are used to determine the amount of other contaminants that may be present in the samples, such as guanidine thiocyanate. The A260/A280 ratios range from 1.9 to 2.1 was acceptable ratios for purity in this experiment, and requirements for A260/A230 ratios are >2.0. RNA concentration were calculated using the 260 nm reading. The Nanodrop spectrophotometer (Thermo Scientific, Waltham, MA, USA) was used to measure absorbance. cDNAs were synthesized by using the RevertAid First Strand cDNA Synthesis Kit (ThermoFisher, Waltham, MA, USA) according to manufacturer’s instructions. The following primer pairs were used: IFN-γ, sense: 5′-CGGCACAGTCATTGAAAGCC-3′ and anti-sense: 5′-TGCATCCTTTTTCGCCTTGC-3′; TNF-α, sense: 5′-ATGAGCACAGAAAGCATGATC-3′ and anti-sense: 5′-TACAGGCTTGTCACTCGAATT-3′; IL-1β, sense: 5′-CAGGATGAGGACATGAGCACC-3′ and anti-sense: 5′-CTCTGCAGACTCAAACTCCAC-3′; IL-37, sense: 5′-TCAGCTGAAGAAGGAGAAACTG-3′ and anti-sense 5′-TTATCTGTCACCCCAACAGG-3′; IL-10, sense: 5′-CTTGCACTACCAAAGCCACA-3′ and anti-sense: 5′-GTTAT GTCTTCCCGGCTGT-3′; GAPDH, sense: 5′-CTCTCTGCTCCTCCCTGT-3′ and anti-sense: 5′-GCAACAATCTCCACTTTG-3′. qPCR amplification reactions were prepared with the SYBR Green PCR Kit (Bio-Rad, Hercules, CA, USA) and performed using the CFX96 Real-Time System (Bio-Rad). Firstly, the amplification efficiency of the target genes are consistent with the GAPDH. Then, each reaction was carried in a total volume of 20 μL including 2 μL cDNA, 10 μL SYBR Premix Ex Taq™ Ⅱ (TaKaRa Biotechnology Co., Ltd., Dalian, China), 0.8 μL/primer and 6.4 μL ddH2O. Amplifcation conditions were: 10 min at 95 °C, followed by 33 cycles of 15 s at 95 °C and 1 min at 60 °C. A melting curve was run to assess the specificity of primers employed. The relative expression level of mRNAs in the colonic tissues was normalized to an internal reference gene GAPDH. Relative expression levels of target genes were calculated by normalization to GAPDH values using the 2^−ΔΔ*C*t^ method.

### 4.6. Immunoblotting

Western blot analysis was performed using standard methods. Briefly, equal amounts of total protein lysates were separated by sodium dodecyl sulfate-polyacrylamide gel electrophoresis (SDS-PAGE) and transferred to a nitrocellulose membrane (Millipore, Billerica, MA, USA) for at least 1 h at 200 mA. Membranes were blocked in 5% non-fat milk in TBST and incubated with primary antibodies against IL-37 (Sigma-Aldrich, St. Louis, MO, USA; 1:1500 in TBST) and β-actin (Abcam, Cambridge, UK; 1:5000 in TBST) overnight at 4 °C. The membranes were then washed for 30 min in TBST and probed with horseradish peroxidase (HRP)-tagged secondary antibodies (ThermoFisher) for 1 hour at room temperature. Membranes were washed again and proteins were visualized with Pierce ECL Western Blotting Substrate (ThermoFisher).

### 4.7. ELISA

Quantitation of cytokines IL-37 in serum was performed by sandwich ELISA using IL-37 Human Uncoated ELISA Kit (Invitrogen, Carlsbad, CA, USA). The sensitivity of detection was 31.3 pg/mL.

### 4.8. Isolation of Splenocytes and Mesenteric Lymph Node Cells (MLNCs) from Colonic Tissue

Mesenteric lymph nodes (MLNs) and spleen were excised. Red blood cells (RBCs) were removed with lysis buffer (Sigma-Aldrich). Single cell suspensions of spleen and MLNs were passed through a sterile wire screen and then were washed twice in RPMI 1640 (Gibco, Life Science, Carlsbad, CA, USA). Cell suspensions were then enumerated using a cell counting chamber and stored in media containing 10% FBS on ice until used within two hours.

### 4.9. Flow Cytometry

Splenocytes and MLNCs were re-suspended in PBS, and viable cells were counted using a solution of 0.4% Trypan blue in PBS. The cells were seeded at a density of 5 × 10^5^ cells per well of a 12-well plate in complete RPMI 1640 containing 1× Cell Stimulation Cocktail (plus protein transport inhibitors) (Invitrogen) and incubated at 37 °C for 16 h. After stimulation, cells were washed in flow cytometry buffer (eBioscience, San Diego, CA, USA) and then were incubated for 30 min with optimal concentration of FITC-conjugated anti-mouse CD3 mAb (eBioscience). To stain for intracellular IL-37, cells were fixed and permeabilized with Cytofix/Cytoperm (BD Biosciences, Sunnyvale, CA, USA) and then stained with PE-conjugated anti-human IL-37 (eBioscience). Cells were washed twice and re-suspended for analysis on a FACSAria II (Becton-Dickinson, Mountain View, CA, USA).

### 4.10. Cell Sorting and Adoptive Cell Transfer

CD3^+^ T-cells were isolated by fluorescence-activated cell sorting (FACS) from splenocytes of IL37^+/+^ mice sacrificed at day 63 following the DSS treatment. Cells were then transferred to wild-type mice (approximately 1 × 10^6^ cells per mouse) by injection via the tail vein followed by the induction of chronic colitis. Control groups were injected with sterile PBS.

### 4.11. Statistical Analysis

A two-tailed Student’s *t*-test was used for the statistical comparison between two groups. One-way ANOVA followed by the Tukey post hoc test for multiple comparisons were used. Results were shown as the mean ± SEM. The software package GraphPad Prism 5 (GraphPad Software, Inc., La Jolla, CA, USA) was used for statistical analysis. Any difference with a *p* value of 0.05 or less was considered significant (* *p* < 0.05; ** *p* < 0.01; *** *p* < 0.001).
